# Analysis of the Predictability of Postoperative Meningioma Resection Status Based on Clinical Features

**DOI:** 10.3390/cancers16223751

**Published:** 2024-11-06

**Authors:** Manfred Musigmann, Burak Han Akkurt, Hermann Krähling, Benjamin Brokinkel, Dorothee Cäcilia Spille, Walter Stummer, Walter Heindel, Manoj Mannil

**Affiliations:** 1 Clinic for Radiology, University Hospital Münster, Albert-Schweitzer-Campus 1, DE-48149 Münster, Germany; burakhan.akkurt@ukmuenster.de (B.H.A.);; 2 Clemenshospital Münster, Department of Neurosurgery, Institute for Neuropathology, University Hospital Münster, DE-48153 Münster, Germany; 3 Department of Neurosurgery, University Hospital Münster, Albert-Schweitzer-Campus 1, DE-48149 Münster, Germanywalter.stummer@ukmuenster.de (W.S.)

**Keywords:** meningioma, postoperative resection status, gross total resection, machine learning, MRI, neuroimaging

## Abstract

**Background:** Our aim was to investigate the predictability of postoperative meningioma resection status based on clinical features. **Methods:** We examined 23 clinical features to assess their effectiveness in distinguishing gross total resections (GTR) from subtotal resections (STR). We analyzed whether GTR/STR cases are better predictable if the classification is based on the Simpson grading or the postoperative operative tumor volume (POTV). **Results:** Using a study cohort comprising a total of 157 patients, multivariate models for the preoperative prediction of GTR/STR outcome in relation to Simpson grading and POTV were developed and subsequently compared. Including only two clinical features, our models showed a notable discriminatory power in predicting postoperative resection status. Our final model, a straightforward decision tree applicable in daily clinical practice, achieved a mean AUC of 0.885, a mean accuracy of 0.866, a mean sensitivity of 0.889, and a mean specificity of 0.772 based on independent test data. **Conclusions:** Such models can be a valuable tool both for surgical planning and for early planning of postoperative treatment, e.g., for additional radiotherapy/radiosurgery, potentially required in case of subtotal resections.

## Simple Summary

This study explored the predictability of meningioma resection status (gross total vs. subtotal) based on clinical features. We analyzed 23 features to determine their effectiveness in forecasting resection outcomes, comparing predictions based on the Simpson grading system and postoperative tumor volume (POTV). Using data from 157 patients, our developed models demonstrated high accuracy with only two key features, achieving an average AUC of 0.885 and accuracy of 0.866. The final model, a simple decision tree, may be useful for guiding decisions for surgical planning and postoperative treatments.

## 1. Introduction

Meningiomas, the most prevalent primary tumors of the central nervous system (CNS), comprise approximately 37.6% of all CNS tumors [1]. Regarding benign brain tumors, meningiomas account for approximately 50%. In the United States, the annual incidence of meningiomas is 5.3 per 100,000 people, increasing steadily with age [2]. These mostly benign, extra-axial tumors originate from the arachnoid cap cells [3]. According to the current WHO classification for CNS tumors from 2021, meningiomas are regarded as a single tumor type with 15 subtypes. Based on histopathology or subtype, meningiomas are classified into the three CNS WHO grades 1–3 [4,5]. About 80–81% of all meningiomas are assigned to CNS WHO grade 1, 17–18% to CNS WHO grade 2, and 1.7% to CNS WHO grade 3 [1,6,7]. Following international guidelines, the primary treatment of meningiomas consists of surgery and adjuvant radiotherapy [8]. Chemotherapy, on the other hand, is only recommended for recurrent or progressive disease if radiotherapy or further surgical resection is not feasible [7].

It has been known for many decades that the extent of resection (EOR) achieved at the first surgical treatment of the tumor has a strong influence on the further prognosis. In his famous work from 1957, Simpson found that the extent of meningioma resection is an important factor in predicting the risk of tumor recurrence [9,10]. The Simpson grading uses five different grades to assess the completeness/incompleteness of tumor resection, dural attachment, and abnormal bone that may be affected. Higher Simpson grades and subtotal resections (STR) are often associated with recurrent tumor growth and thus with the risk of disease progression [11]. Accordingly, the EOR has a significant influence on further treatment planning. It is therefore important to determine the postoperative result as early as possible to be able to plan any further therapeutic steps that may be necessary in a timely manner. Such an early determination of the postoperative resection status is the aim of our study.

In recent years, numerous new methods have been developed in the preoperative diagnosis, prognosis and planning of intracranial meningiomas [12]. For example, in a study conducted by Jimenez et al., machine learning models were developed to predict postoperative outcomes among skull base meningioma patients, prolonged hospital length of stay, nonroutine discharge disposition, and high hospital charges [13]. In many studies nowadays, machine learning is combined with radiomics. Radiomics is a relatively new branch of medical imaging. Many quantitative features are extracted from radiological images such as magnetic resonance imaging (MRI) or computed tomography (CT). Based on this information, it is often even possible to identify disease characteristics that are invisible to the human eye. Machine learning, sometimes in combination with radiomics, enables completely new diagnostic approaches in medicine. For example, various studies have already shown that deep learning and radiomics-based machine learning can be used to determine the WHO grade of meningiomas [14,15,16,17]. The predictability of the postoperative resection status of meningiomas using deep learning has also already been investigated [18]. However, the development of such approaches requires a considerable amount of expert knowledge. In addition, as long as corresponding algorithms have not yet been fully implemented in everyday clinical practice, their use is still associated with a certain amount of effort. In a radiomics-based machine learning model, for example, the area to be examined (i.e., the region of interest) must first be segmented using suitable software; subsequently the radiomic features must be determined and finally, the result/diagnosis must be calculated using an often highly sophisticated model.

Our study explores whether meningioma resection outcomes can be accurately predicted using clinically practical, easily accessible features. For easy clinical application in daily practice, we deliberately avoid tumor segmentation with sophisticated software, radiomic approaches, and deep learning, as performed, for example, by Akkurt et al. [18]. Instead, our analyses exclusively consider readily assessable clinical features. We analyze which features are suitable for making statements about the achievable postoperative resection status. Gross total resection (GTR) and STR are usually defined according to Simpson grading or alternatively according to the postoperative tumor volume (POTV). Simply put, cases in which a meningioma is completely resected are referred to as GTR cases, and cases in which only an incomplete resection is achieved are referred to as STR cases. We analyze three different definitions of GTR/STR and compare the discriminatory power with which the postoperative resection status can be predicted with respect to each of these definitions. Although we use machine learning algorithms such as stepwise logistic regression, Lasso (Least absolute shrinkage and selection operator) regression and random forest for model development, we can subsequently reduce our results to a simple decision tree based on clinical features for the prediction of postoperative outcome, i.e., for the prediction of GTR/STR cases. A decision tree is a tree-like model, built similarly to a flowchart, that can be used to make decisions/predictions based on relevant features. In this way, our final model results can be used very easily in everyday clinical practice without the need for prior tumor segmentation or any calculations, facilitating and accelerating further treatment planning.

## 2. Materials & Methods

Our single center study was performed in compliance with the Declaration of Helsinki [19] and approved by the local ethics committee (Ärztekammer Westfalen Lippe and University of Münster, 2021-596-f-S). Due to the retrospective nature of the study, written informed consent was waived by the Ärztekammer Westfalen Lippe and University of Münster. Our aim is to predict the achievable postoperative meningioma resection status based on clinical features. We analyze the predictability of postoperative resection status, i.e., the distinction between GTR and STR cases in different ways: in terms of the postoperative tumor volume achieved and based on Simpson’s grading. Accordingly, we retrospectively searched our hospital’s database for patients diagnosed with meningioma followed by resection between February 2015 and July 2018. The patient records were screened and evaluated according to their POTV and their Simpson grade. Initially, 165 patients were included. Eight of these patients had to be excluded due to unknown Simpson grade. The final study cohort of 157 patients comprises 110 females and 47 males. Regarding the POTV, a GTR was achieved in 79.6% of all cases (cases with a POTV of zero). In the Simpson grading, grades I to II are sometimes summarized as GTR, but usually grades I to III [20,21,22]. In this case, a GTR was achieved in 77.7% to 83.4% of all cases. The POTV was determined based on contrast-enhanced, T1-weighted postoperative MRI scans. Using the 3D Slicer software (version 5.6.1), the MRI images were segmented semi-automatically and the POTV was subsequently calculated. The Simpson grade achieved was reported by the responsible neurosurgeon. The demographic characteristics of the patients in our study cohort used to predict possible gross total resections based on clinical factors are summarized in Table 1.

A total of 23 clinical features were analyzed regarding their ability to predict postoperative resection status:

(1) Age of the patient at diagnosis
(2) Gender, i.e., distinction between female and male
(3) Distinction between initial diagnosis and recurrence
(4) Karnofsky Performance Scale Index (KPI)
(5) Tumor location
(6) Preoperative tumor volume (in cm³)
(7) Preoperative edema volume (in cm³)
(8) Edema index
(9) Sheeting
(10) Tumor subtype
(11) Distinction between brain invasion yes or no
(12) Distinction between increased cell density yes or no
(13) Distinction between spontaneous necrosis yes or no
(14) Distinction between preoperative epilepsy yes or no
(15) Distinction between other atypia/aplasia criteria yes or no
(16) Distinction between atypia criteria/anaplasia criteria also fulfilled without brain infiltration yes or no
(17) Distinction between regular and irregular shape
(18) Differentiation between T2 intensity yes or no
(19) Distinction between disappeared/disintegrated and intact arachnoid layer T2
(20) Distinction between heterogeneous and homogeneous uptake of contrast agent
(21) Distinction between present and absent calcifications
(22) Distinction between present and absent capsular enhancement
(23) Number of mitoses per 10 HPF (high-power field)

**Figure 1:**
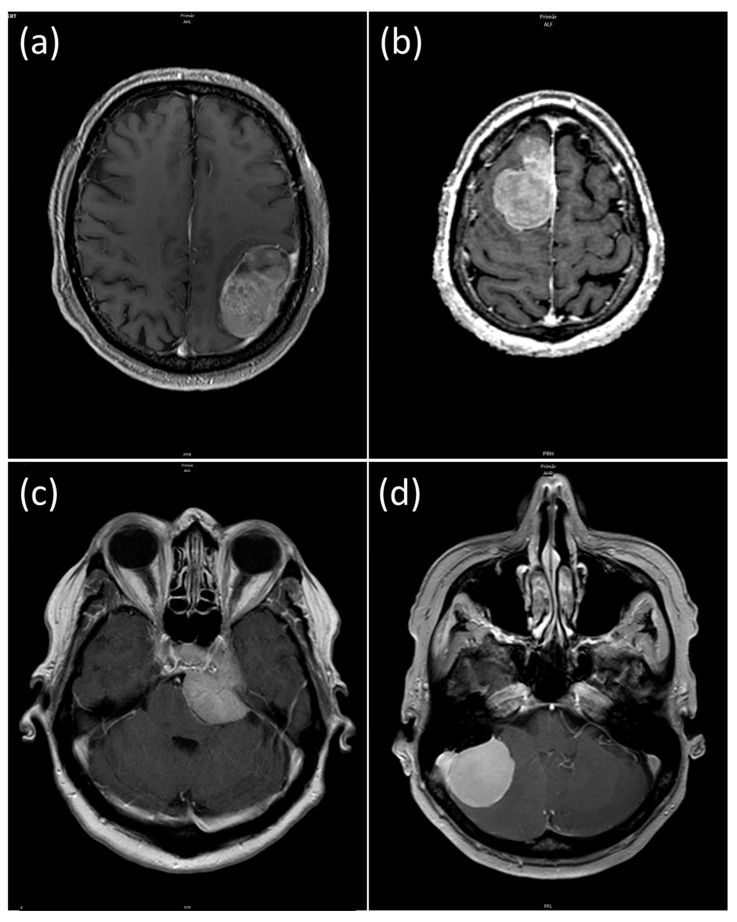
The study cohort contained meningiomas of four different locations: (**a**) convexity, (**b**) falx, (**c**) fossa cranii anterior/media (skull base), (**d**) fossa cranii posterior.

The presence or absence of the above-mentioned clinical and radiological criteria can be used to grade a meningioma; high-grade meningiomas in turn have an increased risk of incomplete resection. For example, radiologic criteria such as regular contour, calcifications and homogeneous enhancement are indicative of a low-grade meningioma, while a larger perifocal edema volume, brain invasion or histologic atypia criteria are indicative of a high-grade meningioma. Moreover, there are criteria that affect surgical management, for example a higher preoperative tumor or edema volume is associated with poorer surgical outcomes. Therefore, it is valuable to summarize all characteristics in an all-encompassing preoperative prediction model. Regarding feature 5, i.e., tumor location, we distinguish between the four locations (a) convexity, (b) falx (c) fossa cranii anterior and media (skull base), and (d) fossa cranii posterior. Figure 1 shows an example of each of these four locations. In our study, we refer to these four different locations (in the order given) as “Location 1 to 4” (e.g., “Location 1” = Convexity, etc.).

**Figure 2:**
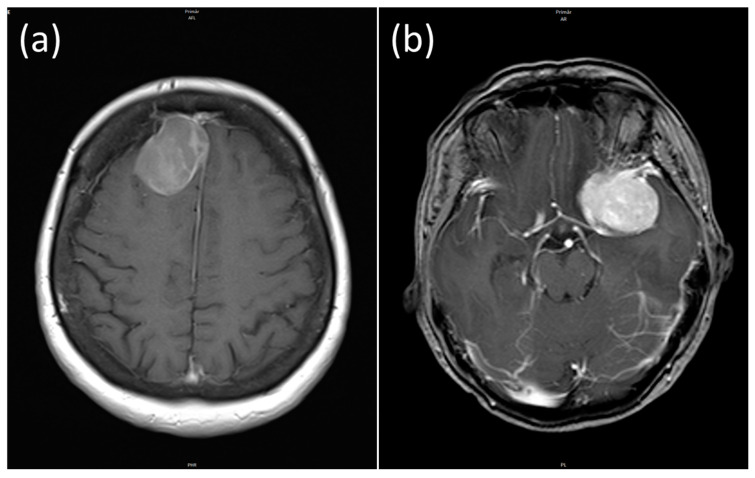
Regular shaped (**a**) and irregular shaped meningioma (**b**) of a 58- and 52-year-old patient.

Sheeting (feature 9) is defined as the absence of a typical meningioma growth pattern. Feature 10 differentiates between the 15 subtypes according to the WHO classification of 2021 (for example “Fibrous meningioma”, “Atypical meningioma”, “Anaplastic (malignant) meningioma” etc.). Feature number 17 distinguishes between meningiomas with a regular and irregular shape. Figure 2a shows a regular shaped meningioma and Figure 2b shows an irregular shaped meningioma.

All categorical features were used in binary form. As an example, we created among others the binary feature “Tumor is located in the convexity? yes or no” based on the feature “Tumor location”. All features were z-score transformed and subsequently subjected to a 95% correlation filter to account for redundancy between the features.

### 2.1. Statistical Analysis

Statistical analyses were conducted using R software (version 4.1.2). Patients were randomly divided into a training cohort and an independent test cohort. A stratified 4:1 ratio was used with a balanced distribution of GTR and STR cases (see Table 1) between the two samples. In predicting postoperative resection status, we analyzed three different definitions in relation to GTR or STR:

Definition “POTV”: GTR: POTV = 0, STR: POTV > 0;

Definition “Simpson 1”: GTR: Simpson grade ≤ II, STR: Simpson grade ≥ III; 

Definition “Simpson 2”: GTR: Simpson grade ≤ III, STR: Simpson grade ≥ IV.

In the following, these three definitions as well as the associated results will be labelled “POTV”, “Simpson 1” and “Simpson 2”. The aim of using these three different definitions for GTR/STR cases is to compare the accuracy with which the postoperative outcome can be predicted in relation to these definitions.

We first analyzed all 23 clinical features examined regarding their univariate discriminatory power and statistical significance. Univariate discriminatory power of the individual features was determined using the Gini coefficient, where the Gini coefficient is calculated as 2*AUC-1 and AUC is the area under the curve (AUC) of the receiver operator characteristic (ROC). The AUC and the Gini coefficient are two of the most important metrics for quantifying the quality (discriminative power) of a model. Perfect models exhibit an AUC and a Gini coefficient of 1. In our case, a value of 1 would imply that the postoperative resection status (GTR/STR) is predicted correctly without exception. The *p*-values were determined to assess statistical significance. Continuous features were first analyzed using the Shapiro–Wilk normality test. Normally distributed features were subsequently analyzed using Bartlett’s test for homogeneity of variances. Normally distributed features with equal variance in the two groups (GTR/STR) were further analyzed using Student’s *t*-test and, in the case of unequal variance, using Welch’s test. Non-normally distributed continuous features, on the other hand, were further analyzed using the Wilcoxon test (Mann–Whitney-U-test). Finally, the chi-square test (Fisher’s exact test) was performed for binary and categorical features. Significance was assumed for all tests results below a threshold value of α = 0.05.

**Figure 3:**
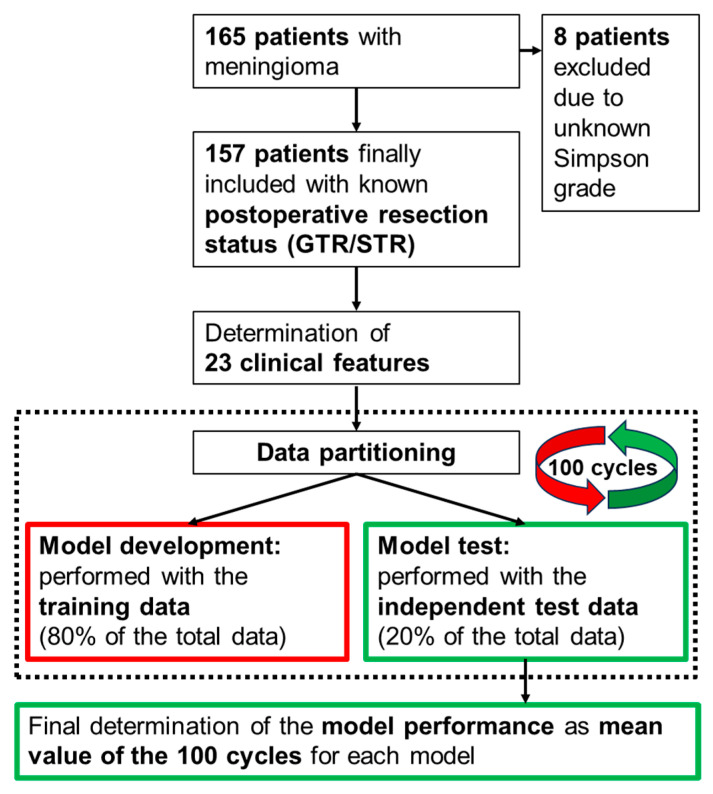
Flowchart describing the methodological approach. For each tested machine learning algorithm (i.e., stepwise logistic regression, Lasso regression, random forest), a total of 10 models are developed with an increasing number (1 to 10) of model features included. Each of these models is developed 100 times, each time using a new data partitioning, and subsequently tested. The final determination of the performance of each model is calculated as the average of the 100 cycles.

Multivariate model development was subsequently carried out using stepwise logistic regression, Lasso regression, and random forest. Feature selection and model optimization were based on the Akaike information criterion (AIC) in case of stepwise logistic regression. In case of Lasso regression and random forest, on the other hand, we used the “varImp” function (varImp = variable importance) for feature preselection, repeated 10-fold cross-validation for hyperparameter tuning and the AUC as well as Cohen’s kappa for model optimization. The complete model developments were carried out exclusively based on the training data. The hyperparameters were optimized using validation data, which in turn are part of the training data. The selection of the utilized model features was also based solely on the training data. Model performance was subsequently determined using the remaining 20% of the total data, i.e., completely independent test data. These independent test data were used completely separately from the training data used previously for the model developments. Since the model performance achieved depends on the number of features contained in the models, we developed our models with an increasing number of features. We started with a one-feature model in each case. The number of features to be included in the final models was determined based on the best AUC achieved. In addition, the model performance achieved also depends on the data partitioning into training and independent test data. For this reason, and to analyze the final model stability, we performed the data partitioning and subsequent full model development and testing for each model 100 times using 100 different data partitions. All performance values of the models were calculated as the mean values of these 100 runs. In addition, the respective 95% confidence intervals were determined. For better understanding, the entire process, consisting of data filtering, data partitioning, model development using 100 repetitions as well as the subsequent model testing, is summarized in a flow chart in Figure 3.

Model performance was determined based on AUC, accuracy, Cohen’s kappa, sensitivity, specificity, positive predictive value (PPV) and negative predictive value (NPV). The accuracy describes the proportion of correctly predicted cases overall, the sensitivity of the proportion of correctly predicted GTR cases, and the specificity the proportion of correctly predicted STR cases. The PPV indicates the ratio of correctly predicted cases with a GTR in relation to all predicted cases with a GTR and the NPV the corresponding ratio in relation to the STR cases. Finally, Cohen’s kappa is calculated as kappa = (observed accuracy − expected accuracy)/(1 − expected accuracy)). Given the large imbalance in our GTR/STR class distribution of more than 3:1 (see Table 1), Cohen’s kappa provides a more objective description of model performance than accuracy. For unbalanced class distributions, higher, i.e., better values (closer to the +1 value) are much more difficult to achieve for Cohen’s kappa than for accuracy.

### 2.2. Results

We started our analyses by first calculating the univariate discriminatory power and *p*-values (significance) for all clinical features investigated (listed in Section 2). As described above, binary features were generated from all categorical features with more than two possible categories. Table 2 lists all features with a significant *p*-value < 0.05 in relation to at least one of the three analyzed definitions for postoperative resection status (labelled as “POTV”, “Simpson 1” and “Simpson 2”). The table also contains the corresponding univariate power values, calculated as Gini coefficients.

The different possible tumor locations are particularly important for predicting the postoperative resection status. The feature “Location 1” describes tumors in the convexity, “Location 2” tumors in the falx, “Location 3” tumors of the fossa cranii anterior and fossa cranii media (skull base) and finally “Location 4” tumors of the fossa cranii posterior. The feature “Location 1 and 2” combines the two locations 1 and 2 and distinguishes them from meningiomas that are assigned to the cranial fossa (as a whole, i.e., location 3 or 4). The feature “Shape”, which differentiates between regular and irregularly shaped meningiomas (see Figure 2), also shows a very high univariate discriminatory power. Regarding Simpson grading, the edema volume and the edema index exhibit a certain discriminatory power. In contrast to the edema volume, the tumor volume was slightly below the significance threshold and is therefore not listed in Table 2. In addition, the distinction between patients with a KPI > 80 and patients with a KPI ≤ 80 also showed a certain degree of discriminatory power. However, this only applies in relation to the POTV definition for the postoperative resection status. Finally, the last feature (labelled “Ini_diag_vs._recur”) distinguishes between an initial diagnosis and a recurrence. The univariate discriminatory power of this feature is limited and correspondingly close to the significance threshold of 0.05.

**Figure 4:**
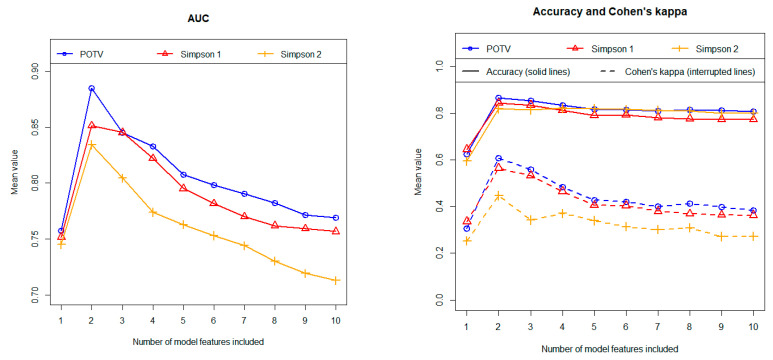
Prediction of postoperative resection status (GTR/STR) using stepwise logistic regression. Left figure: area under the curve (AUC). Right figure: accuracy and Cohen’s kappa. All results were calculated using independent test data and as mean values of 100 repetitions (100 cycles). Three different definitions regarding the postoperative outcome were tested in terms of their predictability (as indicated in the figures).

**Figure 5:**
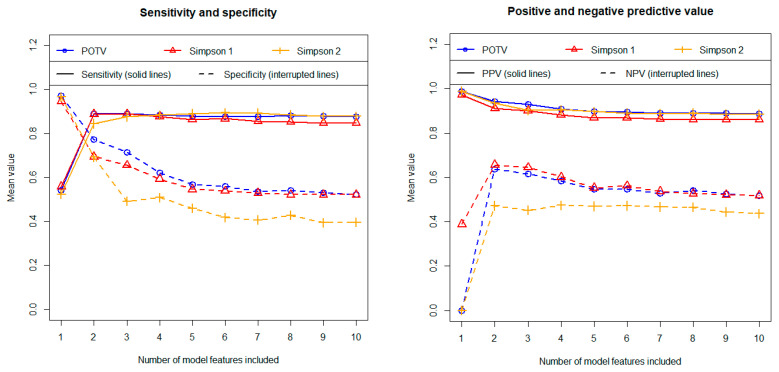
Prediction of postoperative resection status (GTR/STR) using stepwise logistic regression. Left figure: sensitivity and specificity. Right figure: positive predictive value (PPV) and negative predictive value (NPV). All results were calculated using independent test data and as mean values of 100 repetitions (100 cycles). Three different definitions regarding the postoperative outcome were tested in terms of their predictability (as indicated in the figures).

Following the univariate analyses, we developed multivariate models. The three algorithms analyzed, i.e., stepwise logistic regression, Lasso regression and random forest, demonstrated comparable discriminatory power based on independent test data. Overall, however, the stepwise logistic regression performed best. Figure 4 shows the results for both AUC (left figure) and accuracy and Cohen’s kappa (right figure) obtained with stepwise logistic regression in relation to the three different definitions regarding the postoperative outcome. The associated calculation results for the sensitivity, the specificity as well as for the positive and negative predictive value are summarized in Figure 5. The results were calculated using independent test data and are shown as a function of the number of features included. As described above, all results were determined as mean values of 100 repetitions using 100 different data partitions.

The model predicts postoperative resection status most accurately when using the postoperative tumor volume (POTV) definition. In contrast, our calculations provide the least precise results for the widely used definition, where STR cases are defined as cases with a Simpson grade ≥ IV (labelled “Simpson 2” in the figures). Finally, the definition in which resections with a Simpson grade III are regarded as STR cases (“Simpson 1”) results in a discriminatory power that is only slightly lower than those obtained with the definition based on the postoperative tumor volume.

Regarding most metrics (i.e., AUC, accuracy, Cohen’s kappa, sensitivity and NPV) two-feature models yielded the highest predictive values. The discriminatory power values obtained on average over 100 cycles (including the 95% confidence intervals) are summarized for the 2-feature models in Table 3.

As described, the models were each completely developed 100 times using 100 different data partitions. Accordingly, the 100 associated models may differ in their feature composition. We therefore analyzed how stable the models are in terms of their feature composition. The feature “Shape” was selected in 100% of all runs in the cases of the two definitions “POTV” and “Simpson 1” regarding the postoperative resection outcome and in 99% of the runs in the case of the “Simpson 2” definition. The second important feature is “Location 1 and 2”. This feature was selected in 99%, 99%, and 97% of all runs. Thus, the feature composition of the models, containing only two features, proves to be extremely stable. The third most important feature was the preoperative tumor volume. This feature was selected in 98%, 84%, and 71% of the runs. On average, however, the inclusion of the tumor volume in the models did not improve the discriminatory power (see Figure 4 and Figure 5). This means that only the two features “Location 1 and 2” and “Shape” contribute to the discriminatory power in the multivariate models. These are exactly the two features with the highest Gini coefficients in the univariate analyses (see Table 2).

**Figure 6:**
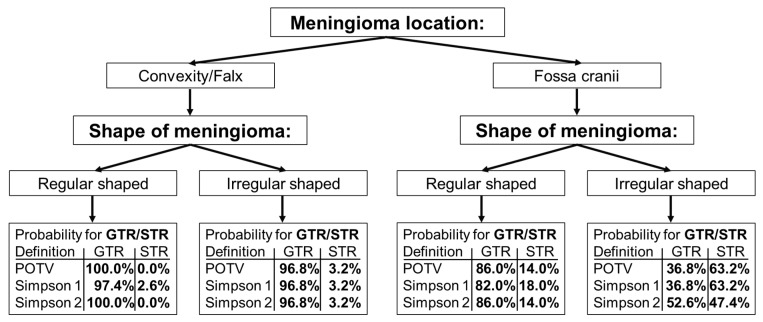
Decision tree for predicting the postoperative resection status (GTR/STR) in meningiomas. Three different definitions regarding the classification of postoperative outcomes were examined: “POTV”: GTR are cases with a postoperative tumor volume = 0; “Simpson 1”: GTR are cases with a Simpson grade ≤ II; “Simpson 2”: GTR are cases with a Simpson grade ≤ III. The percentage values in the figure indicate the probabilities for the associated postoperative outcome.

Based on the two most important features, we developed a simple decision tree to predict postoperative resection status (Figure 6). The decision tree contains the respective probabilities for a GTR/STR for the three definitions analyzed in relation to the postoperative resection status.

The decision tree indicates that regularly shaped meningiomas in the convexity and falx regions are typically amenable to complete resection. The STR probability is only slightly increased for irregularly shaped meningiomas regarding these brain regions. Meningiomas located in the fossa cranii exhibit an increased risk for a subtotal resection. This applies in particular to irregularly shaped meningiomas. This last group of meningiomas has a very high probability of 63.2%, 63.2% and 47.4% for STR with respect to the three studied definitions regarding the postoperative resection outcome.

It should be noted that this decision tree, which in contrast to the results shown in Table 3 contains the two features “Location 1 and 2” and “Shape” in a fixed form, has a very similar discriminatory power to the models previously created with variable feature composition. For example, based on the independent test data, the following results for the AUC were obtained with respect to the three definitions examined (“POTV”, “Simpson 1”, “Simpson 2”): 0.886 [0.717:0.980], 0.853 [0.670:0.989] and 0.847 [0.687:0.962]. Without exception, the results obtained in relation to the other metrics were also very close to the values given in Table 3. This again confirms the model stability, which was previously already evident in the feature composition.

## 3. Discussion

In our study, we investigated the predictability of the postoperative meningioma resection status based on clinical features. We analyzed and compared three common definitions of the postoperative resection status. As we have shown, the postoperative resection outcome can be predicted with high accuracy based on a simple decision tree. In contrast to many models, which often require sophisticated software and a high level of expert knowledge, our results are very easy to apply in daily clinical practice. We analyzed a total of 23 important clinical features and showed which of these features are suitable for making a preoperative statement about whether a complete or only a subtotal meningioma resection can be achieved. The differentiation between regular and irregularly shaped meningiomas as well as the tumor location have proven to be extremely suitable for predicting the postoperative resection status. Our results regarding tumor location are consistent with the findings of other studies. Lemée et al. analyzed risk factors for incomplete resection. In line with our results, they found that a tumor location at the skull base is one of the most important risk factors [23]. In their publications on surgical experiences, both Lobato et al. and Roberti et al. stated that meningiomas of the fossa posterior are often difficult to resect completely [24,25]. According to our database, about one-third of fossa cranii meningiomas (anterior, media, and posterior) could only be subtotally resected. Conversely, STR cases in convexity and falx regions comprised only 1% to 3%.

In our analyses, we found that the postoperative resection outcome can be predicted accurately in terms of the postoperative tumor volume. In comparison, the predictability of GTR/STR based on Simpson grades was slightly worse. This may be partly due to the fact that POTV can be determined accurately and reproducibly using postoperative MRI imaging, whereas the Simpson grade assigned by the neurosurgeon may have a certain subjective component. It is interesting to note that we were able to better predict the postoperative resection status of Simpson grade III meningiomas if these cases were classified as STR rather than GTR cases. In our database, six of nine patients with a Simpson grade III had an irregularly shaped meningioma located at the skull base. In this constellation, our results indicate that it is likely that only a STR can be achieved. Accordingly, most of our cases with a Simpson grade III would be categorized as STR with respect to our data. However, in contrast to our results, grade III meningiomas are usually still classified as GTR cases. Brokinkel et al. also analyzed the optimal threshold for gross total resection in meningioma surgery in relation to the Simpson grading. They conclude that the value for the prediction of progression/recurrence is higher when dichotomizing into Simpson grade I–III vs. ≥ IV than into grade I–II vs. ≥ III resections [26]. Chotai et al. suggest that the Simpson grade should no longer be of such great importance in modern meningioma surgery. In line with our results, they discuss whether the classification as GTR/STR should be replaced by a grading scale that relies on postoperative MRI imaging considering the residual tumor volume [21].

As early as 1957, Simpson already showed that the extent of surgical resection and tumor recurrence are correlated [9]. His findings are still valid today. For example, Voß et al. showed that increasing Simpson grades or subtotal resections remain correlated with tumor recurrence [10]. Wang et al. also found that the extent of surgical resection of meningiomas at the skull base significantly influences prognosis. Gross total resection of meningiomas improved progression-free survival compared to subtotal resection [27]. Gallagher et al. concluded that the Simpson grade is still a predictive factor for recurrence and progression-free survival. In addition, they analyzed that the location of the meningioma no longer appears to have a significant effect on progression-free survival. As they suspect, this is due to the increased use of adjuvant therapies, as well as advances in technology and surgical techniques [28]. Przybylowski et al. analyzed recurrence-free survival (RFS) in relation to Simpson resection grades. They suggest that, when feasible, Simpson grade I resection should remain the goal of intracranial meningioma surgery. They found that Simpson grade IV resection with adjuvant radiosurgery resulted in similar RFS compared with Simpson grade II and III resections [29].

As the results of the above-mentioned studies show, it is important for further treatment planning to be able to predict the postoperative resection status as early as possible. In this way, further treatment steps, such as subsequent radiotherapy, if necessary, can be planned quickly. The EOR achieved is also of great importance for the frequency of the necessary follow-up patient monitoring.

Our proof-of-principle study has several limitations that need to be considered. For clinical application, it should first be mentioned that further information about the patients would be beneficial. Two further important factors not included in our database were identified by Lemée et al.: symptoms at presentation (seizure, intracranial hypertension and/or a neurological deficit) and associated bone invasion [23]. Corniola et al. specifically examined the resectability of posterior fossa meningiomas. They also found bone invasion to be an important predictive factor [30]. It would also be very important to consider whether important vessels (sinuses) or nerves are located near the meningiomas or are even involved. For example, meningiomas involving the cavernous sinus are almost never resected completely. Proximity to neurovascular structures often poses a problem/risk during meningioma resection. Also, the neurosurgeon and their experience certainly influence the extent of the resection achieved. Finally, the specific goal of the operation, such as a decompression of the brainstem, has a significant influence on the resection result. If such further important information were also present, the decision tree we presented could be further refined. Subsequently, it would probably be possible to make even more accurate predictions. In addition to the previously mentioned options for further improving clinical applicability, the retrospective study design and the size of the study cohort should also be mentioned as limitations. Furthermore, the study is based on a single-center data set. These limitations could lead to bias, and the study cohort may have limited representativeness for other patient collectives. Despite these limitations, our decision tree-based model shows very promising results for predicting the postoperative resection status and is very easy to use in clinical practice.

## 4. Conclusions

Postoperative resection status in meningiomas can be accurately predicted using a small set of clinical features, notably tumor shape and location. Particularly important is, on the one hand, the distinction between meningiomas with a regular and an irregular shape and, on the other hand, the location of the tumor. We developed a model that can easily be integrated into everyday clinical practice. Such methods can speed up and simplify therapy planning. However, to be able to further increase the accuracy of our model, larger patient cohorts and further important clinical information, such as knowledge of the involvement of important vessels, would be beneficial.

## Figures and Tables

**Table 1:** Demographic characteristics of the patients in our meningioma study cohort for predicting postoperative resection status based on clinical features.

	Training Data	Independent Test Data	Total Data
Number of patients	126	31	157
Gender (in %)			
Female	69.84	70.97	70.06
Male	30.16	29.03	29.94
Mean age (in years)	60.15	59.82	60.08
Postoperative tumor volume (in %)			
=0 (GTR)	79.37	80.65	79.62
>0 (STR)	20.63	19.35	20.38
Simpson grade (in %)			
I (GTR)	28.50	29.32	28.66
II (GTR)	49.05	49.03	49.04
III (GTR or STR)	5.71	5.84	5.73
IV (STR)	15.55	14.23	15.29
V (STR)	1.20	1.58	1.27

**Table 2:** Univariate discriminatory power (Gini coefficient) and *p*-values for all features with a significant *p*-value < 0.05 in relation to at least one of the three definitions analyzed.

	POTV		Simpson 1		Simpson 2	
Feature	Gini (in %)	*p*-Value	Gini (in %)	*p*-Value	Gini (in %)	*p*-Value
Location 1	39.2	<0.0001	32.8	0.0005	32.8	0.0022
Location 2	12.1	0.1258	16.4	0.0228	15.3	0.0702
Location 3	38.9	0.0002	38.6	0.0001	40.3	0.0004
Location 4	13.2	0.0405	11.4	0.0702	8.5	0.2939
Location 1 and 2	51.3	<0.0001	49.2	<0.0001	48.1	<0.0001
Shape	42.9	<0.0001	35.4	0.0004	34.9	0.0022
Edema volume	18.0	0.0753	25.2	0.0099	24.6	0.0246
Edema index	16.9	0.0950	23.3	0.0171	22.6	0.0390
KPI > 80	24.1	0.0241	14.1	0.1961	17.2	0.1605
Ini_diag_vs._recur	14.7	0.0339	12.6	0.0630	15.4	0.0431

**Table 3:** Prediction of postoperative resection status (GTR/STR) using stepwise logistic regression. Results in relation to the different metrics for the models with two features. All results were calculated using independent test data and as mean values of 100 repetitions (100 cycles). The numbers in brackets indicate the 95% confidence intervals.

Metric	POTV	Simpson 1	Simpson 2
AUC	0.885 [0.717:0.980]	0.851 [0.670:0.989]	0.834 [0.580:0.962]
Accuracy	0.866 [0.742:0.968]	0.844 [0.710:0.983]	0.820 [0.662:0.935]
Cohen’s kappa	0.608 [0.250:0.903]	0.565 [0.210:0.954]	0.448 [0.022:0.795]
Sensitivity	0.889 [0.760:1.000]	0.888 [0.792:1.000]	0.844 [0.692:0.962]
Specificity	0.772 [0.333:1.000]	0.694 [0.429:1.000]	0.692 [0.200:1.000]
PPV	0.943 [0.849:1.000]	0.911 [0.826:1.000]	0.936 [0.846:1.000]
NPV	0.638 [0.368:1.000]	0.657 [0.375:1.000]	0.472 [0.221:0.716]

## Data Availability

The datasets used and/or analyzed during the current study are available from the corresponding author on reasonable request.
